# The Perspective of Romanian Patients on Continuous Therapy for Multiple Myeloma

**DOI:** 10.3390/jpm14090910

**Published:** 2024-08-28

**Authors:** Ruxandra Irimia, Sorina Nicoleta Badelita, Sinziana Barbu, Larisa Zidaru, Ioana Loredana Carlan, Oana Diana Preda, Daniel Coriu

**Affiliations:** 1 Department of Hematology, “Carol Davila” University of Medicine and Pharmacy, 030167 Bucharest, Romania; 2 Fundeni Clinical Institute, 022328 Bucharest, Romania

**Keywords:** continuous therapy, multiple myeloma, patient perspective, quality of life

## Abstract

The treatment paradigm of multiple myeloma (MM) has shifted in the past years, as continuous therapy is becoming the standard of care for both newly diagnosed and relapsed patients. Although it is indisputable that continuous therapy has added a great benefit on the progression-free as well as overall survival, it is still unclear what the patients’ perspective is on this therapeutic approach. Methods: This study included 155 adult MM patients from Fundeni Clinical Institute in Romania, receiving continuous therapy with daratumumab, proteasome inhibitors, immunomodulators, or bi-specific antibodies. The patients had varied economic, social, and educational backgrounds. We developed a questionnaire to interrogate the quantitative and qualitative effect of the therapy on the patients’ personal and professional life and to identify the side effects that had the strongest impact on their quality of life. Results: 74.83% of the patients reported that the treatment they received negatively impacted their quality of life. Among them, 40% considered that the most detrimental aspects of the therapy are the financial burden and the negative impact on their professional life. One-third of the patients reported that the therapy negatively impacted their personal life and that it had a deleterious effect on their relationship with their partner and family members. In terms of the side effects experienced, patients considered that tiredness was the main factor causing a decrease in their quality of life, followed by insomnia and bone pain. Despite this, almost none of the patients considered dropping the therapy, and almost half of the patients considered that the frequent visits to the hospital offered them psychological comfort. In addition, more than 70% of the patients declared that they were afraid to stop the therapy if given the choice, with the main concerns being the fear of an early relapse. Conclusions: Although continuous therapy is associated with a high financial burden and a negative impact on both professional and personal life, the frequent visits to the hospital appear to be reassuring. Moreover, the patients would not opt for treatment discontinuation and felt safer when monitored frequently.

## 1. Introduction

The overall survival (OS) of multiple myeloma (MM) has improved substantially in the past decade due to the emergence of new antimyeloma drugs [1,2,3]. Currently, the treatment paradigm has shifted from limited-duration therapy towards continuous therapy [4]. The latest treatment regimens, incorporating proteasome inhibitors (PIs), monoclonal antibodies, and immunomodulatory drugs (IMIDs), are now administered as induction or maintenance therapy until disease progresses or significant toxicity occurs. This treatment approach has enhanced disease control, providing deeper responses, prolonged progression-free survival, and delayed relapses [5,6,7,8]. Moreover, the large number of therapeutic options offers the chance of delivering a more tailored therapeutic approach, rather than the conventional “one size fits all”.

Traditionally, various disease and patient characteristics, such as cytogenetics, stage, presence of extramedullary disease, previous lines of therapy, age, and comorbidities, are factored in when selecting a treatment regimen. However, the rate of response, progression-free survival, and overall survival are no longer the main determinants of treatment success in chronic malignancies. Patients who undergo prolonged treatments for incurable cancers often feel that they are trading their quality of life (QoL) for duration of life [9]. QoL encompasses patients’ perspective of the disease and therapy impact on their physical, psychological, and social wellbeing [10]. 

Considering that MM has become a chronic disease with a 70% median 5-year survival, it is becoming increasingly clear that the patient’s perspective, QoL, and overall satisfaction should be equally important factors in therapy selection [11,12,13]. The patient’s perspective should be recognized as an important determinant of overall architecture of myeloma management, as it directly affects compliance, treatment adherence, satisfaction with the medical care, and, eventually, the success of the medical intervention [14].

Despite tremendous therapeutic advancements in MM, there is still limited knowledge about patients’ perspectives on continuous therapy. Acknowledging the significance of patient perspectives, our study aims to address this gap in knowledge by exploring the impact of continuous therapy on patients’ day-to-day life. These findings are particularly relevant when selecting a treatment regimen, especially in countries like Romania, where patients often face logistical challenges such as long weekly commutes for treatment. Frequent hospital visits for treatment not only impose financial burden, but can also have significant impact on patients’ physical, psychological, and social wellbeing.

## 2. Methods

Study design and participants: This study aimed to evaluate patient perspectives on continuous therapy for multiple myeloma, focusing on factors previously identified as important determinants such as familial, professional, financial, social, and psychological wellbeing or side effect perception [11,15]. Patients were recruited from Fundeni Clinical Institute, one of the largest public hospitals in Romania serving as a major referral center. Fundeni is also the main tertiary hematology center in Romania, where over half of all the MM patients in our country are diagnosed and/or treated. Adult MM patients receiving continuous therapy for at least 6 months prior to the study commencing were eligible for inclusion. Patients receiving treatment either in the outpatient department or through short admissions (<72 h) were considered. Exclusion criteria comprised critically ill patients or those with impaired mental status. Patients who discontinued therapy due to progressive disease or adverse events prior to the study initiation were also excluded. We identified 168 patients treated with regimens based on monoclonal antibodies (daratumumab), proteasome inhibitors (carfilzomib, ixazomib), immunomodulatory drugs, and bispecific antibodies (teclistamab) either as the first-line therapy or in the relapsed setting. We excluded 13 patients from the final analysis due to incomplete completion of the questionnaire. The data were collected over the time of five months, between March and July 2023. All the patients provided their informed consent to participate in the study.

Questionnaire development: A questionnaire was developed to assess the impact of continuous therapy on various aspects of patients’ lives using both qualitative and quantitative instruments. 

The qualitative questions used a 5-point linear scale (none, low, medium, high, very high) to evaluate the impact of the therapy on different aspects of the patients’ lives. The quantitative questions relied on yes/no responses. The questionnaire was developed using questions from the existing validated quality of life, disease burden, and anxiety scales (EORTC QLQ-C30, EORTC QLQ-MY20, Generalized Anxiety Disorder 7-item scale). The levels of anxiety associated with the upcoming hospital visit were defined as “fear that something bad will happen”, “worry”, “nervousness”. The patients were interrogated regarding their opinion about the most frequently encountered adverse events (infections, fatigue, bleeding risk, bone pain, stool changes) using the question model proposed by Janssens et al. [14]. However, our previous experience utilizing these scales among patients with diverse educational backgrounds revealed response biases. Patients often sought assistance from the medical team to comprehend and complete the questionnaire. This proximity to medical staff led patients to censor their negative perceptions regarding therapy. Moreover, the standard QoL questionnaires typically assess the presence or absence of specific symptoms or the general health status, leaving out patients’ beliefs or attitudes towards treatment, which we specifically wanted to capture in our study.

In our questionnaire, the questions were, therefore, translated and adapted to mitigate response biases; simplified language and accessibility were prioritized to accommodate patients with diverse educational backgrounds. The design of the questionnaire was a collaborative effort between the Fundeni Clinical Institute Multiple Myeloma group and the Romanian MM patient association, SOS Myeloma. 

Five of the seven study members, who were not directly involved in adapting the questions, validated the content of the questionnaire. These physicians were asked to categorize each question as nonrelevant, relevant but not essential, or essential. Only the questions deemed essential were retained in the final version of the questionnaire. Representatives from the patient association served as a pilot group of respondents. We used this pilot phase to assess the face validity of the study, evaluating the questions for clarity, lack of ambiguity, absence of leading or double-barreled questions, sentence structure, difficulty level, and overall appropriateness.

The questionnaire aimed to gather insights into the psychological wellbeing, financial wellbeing, family life, relationship with their partner, and the ability to develop future plans. Additionally, to address our specific research objectives, we included questions related to patient preferences for short-term therapy versus continuous therapy and perception on continuous treatment. The questions were provided in accessible Romanian language, with a Flesch–Kincaid grade level of approximately 7th grade, and reading ease score of 63.65. 

Data collection: Questionnaires were administered in person during hospital visits, ensuring privacy and anonymity in order to encourage open expression of patient concerns. Basic demographic information was collected, including age, sex, education level, profession, and time since MM diagnosis, alongside questionnaire responses for subsequent analysis.

Statistical analysis: DataTab was used to perform the statistical analysis. The chi-square test was performed to investigate any significant differences between groups of participants. A *p* value of <0.05 was considered significant. 

Ethical approval: This study received approval from the Fundeni Clinical Institute ethical committee (Reference 54223), ensuring that it adhered to ethical guidelines for research involving human participants.

## 3. Results

Demographic characteristics: The study included a cohort of 155 patients diagnosed with multiple myeloma (MM) undergoing continuous therapy. Among these participants, 86 individuals (55.48%) were males. In terms of age distribution, the majority, constituting 56.77% of the patients, were below the age of 65, while 43.22% were aged 65 years and above. As for educational backgrounds, a substantial proportion of patients (61.93%) had completed undergraduate studies; 17.41% of patients held primary school degrees, and 44.51% had graduated from high school. A considerable segment, comprising 38.06% of patients, had attained university or post-university qualifications.

In the context of occupational status, 103 patients (66.45%) were retired, either due to advanced age (69.90%) or due to the negative impact of the disease on their ability to work (30.10%). A total of 28.38% of the patients were still professionally active, while the remaining patients reported having no occupation. 

In the study cohort, approximately half of the patients had received their diagnosis more than 3 years prior. A significant subset, representing 30.32% were diagnosed between 1 and 3 years ago, while 18.70% of patients had been diagnosed less than 1 year prior, but had received at least 6 months of therapy at the moment of the questionnaire. The detailed patient characteristics can be found in Table 1.

Treatment impact and preferences: When questioned about the treatment impact on their overall QoL, 74.83% of the patients perceived a negative impact of the continuous MM therapy on their wellbeing, with no statistically significant differences between patients diagnosed less than 1 year prior or 1–3 years prior versus patients diagnosed >3 years prior (66.67%, 74.29%, 75%, *p* = 0.2904 and *p* = 0.715, respectively).

Given the option between high-intensity fixed-duration therapy versus continuous therapy, 59.35% from the total number of patients would prefer a fixed-duration therapy if the overall efficacy would match the continuous therapy. Out of these patients, only 28.26% would still select the fixed-duration therapy if the overall efficacy was inferior to the continuous therapy. In addition, when asked whether they were afraid to discontinue the continuous therapy, 73.54% of the participants expressed their concern, primarily due to fear of a more rapid or more aggressive disease relapse. Moreover, despite the reported negative effects of the continuous therapy on the various aspects of their lives, only 6.45% of the patients had ever considered dropping out of therapy.

Impact on psychological wellbeing: We defined anxiety as “fear that something bad will happen”, “worry”, or “nervousness” concerning their upcoming hospital visit. In general, among the chronically ill population, hospital visits can be accompanied by variable levels of anxiety. The reasons behind this comprise the feeling of uncertainty, the long waiting times, fear of invasive medical procedures, complications, or relapse, and also the disruption to their daily routine. Also, most of the MM patients need to travel frequently across the country to our hospital to receive treatment, due to poor availability of modern drugs in the local hospitals or the absence of a local hematology department altogether. Despite this, in our study group, only 18.06% of the patients reported a high or very high level of anxiety associated with hospital visits. Surprisingly, patients that had been diagnosed less than one year before and received therapy at least in the past 6 months had lower levels of anxiety compared to patients diagnosed between 1 and 3 years prior or those diagnosed over 3 years prior, although the differences were not statistically significant (6.89%, 18.18%, 22.78%, *p* = 0.17, *p* = 0.06, respectively). 

Although tiring, the contact with the medical staff and their physician can be a source of reassurance for some patients, and in our cohort, 43.87% of the patients considered that the frequent hospital visits offered them a high or very high level of psychological comfort, while only 23.87% reported having no or low psychological comfort.Subsequently, we interrogated whether the perception of psychological comfort offered by frequent hospital visits was influenced by the period of time elapsed since diagnosis. Indeed, for newly diagnosed patients, the frequent hospital visits offered psychological comfort in only 24.13% of the cases, while for patients that had over 3 years since diagnosis, the hospital visits were reassuring for 51.28%, *p* = 0.012.

We also observed a statistically significant difference in the reported level of psychological alleviation between the participants that had a university/post-university degree (35.59%) versus participants that graduated from primary school/high school (50%) (*p* = 0.08).

Financial and professional impact: When questioned about the financial impact of the treatment, 36.12% of the patients reported that the continuous therapy has had a very high or high impact on their economic status. There was no statistical difference in the reported financial impact between the patients that were retired (39.81%) versus professionally active (27.27%) (*p* = 0.17) or between patients younger than 65 years versus 65 and over (*p* = 0.547). One-fifth of the patients reported that the treatment interfered at a high or very high level with their professional life. Not surprisingly, the patients that were still professionally active were significantly more impacted by the therapy compared to retired patients or patients without occupation (40.90% versus 11.71%, *p* = 0.022).

Familial and social impact: Regarding their personal life, a quarter of the patients reported that myeloma therapy had a high or very high negative impact on their family life wellbeing, while 31.61% considered that the treatment was negatively impacting their family members. The same percentage of responders considered that their relationship with their partner had been affected by the therapy. There was no statistically significant difference between males and females regarding their perception on the therapy’s negative impact on family life and relationship. A total of 17.41% of the patients reported that continuous therapy has had a high or very high negative impact on their social life. There were no statistically significant differences between patients under 65 years versus patients aged 65 or older. Overall, 31.61% of patients in our study group reported that the therapy interfered with their capacity for making long-term plans. Surprisingly, patients aged 65 or older reported significantly higher levels of distress caused by the inability to make long term plans compared to younger patients under 65 years (73.13% vs. 30.68%, *p* < 0.01). 

Symptom perception: The most burdening symptom was fatigue, which had a high or very high negative impact on 54.19% of the patients, followed by insomnia in 42.58% of the responders. A total of 38.70% of the patients regarded bone pain as highly or very highly bothersome. 

Out of the total, 29.67% of patients indicated that infectious events had a very high or high negative impact on their day-to-day life. Bleeding events were considered relevant by 21.93% of patients, surpassed by stool changes in 30.32% of the patients.

We did not observe any statistically significant differences in the reported burden of symptoms between patients diagnosed less than one year prior, versus 1–3 years prior, versus more than 3 years prior. Also, the type of the therapy backbone did not determine any statistically significant differences in the reported symptoms.

**Figure 1:**
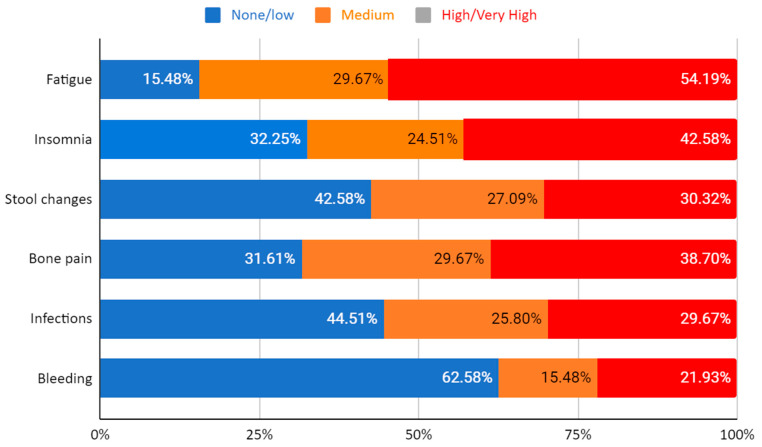
Patient perspectives on symptoms associated with continuous therapy.

The graphic representation of the symptoms is detailed in Figure 1.

## 4. Discussion

The concept of continuous therapy in multiple myeloma was initially validated through the use of interferon maintenance post ASCT, which resulted in an improved PFS [16]. Building on these early efforts, the advent of new antimyeloma agents with improved safety profiles has paved the way for the development of extended duration maintenance regimens and continuous induction treatments, intended for both newly diagnosed and relapsed MM patients. The efficacy of these strategies in improving the outcomes of MM patients has been substantiated in numerous clinical trials and further validated in real-world settings [5,7,17,18,19,20,21,22,23]. As continuous therapy offers unprecedented survival rates, it is becoming increasingly clear that further efforts need to be made to understand the long-term impact on the patients’ overall physical, emotional, professional, and social wellbeing. 

Traditionally, therapy selection has prioritized disease-specific and patient-related factors, such as stage, cytogenetics, age, and comorbidities. Efforts have been also made towards personalizing therapy using pharmacogenetics, single-cell sequencing, or single-cell transcriptomics [24,25,26]. However, the patient’s perspective must be acknowledged as a crucial factor in therapy selection due to its direct impact on treatment adherence and satisfaction, and the overall success of medical interventions [14]. Often, a physician’s perception of the most appropriate therapy may not align with the patient’s preference. Many studies report that chronic oncology patients feel compelled to sacrifice QoL for extended survival [9]. In chronic diseases, successful management relies on the fine balance between optimal disease control, symptom alleviation, side effects, and impact on the patient’s day-to-day life [27].

The existing studies reported that MM is associated with the highest level of symptoms and the lowest levels of QoL among the hematological cancers [12]. Under these circumstances and with current improved survivorship, it is important that the chosen treatment regimen does not further decrement the QoL [13,14,15]. Nevertheless, the improved progression-free survival (PFS) in multiple myeloma does not always correlate with an increase in QoL.

A recent analysis on the reported QoL results from the ASPIRE, ENDEAVOR, and CASTOR and POLLUX clinical trials, all of which administered continuous therapy, showed a consistent reduction in the QoL status over time [28]. All four clinical trials used the EORTC QLQ-C30 and its subscales to evaluate the reported outcomes. The ASPIRE clinical trial, evaluating the efficacy of carfilzomib–lenalidomide–dexamethasone compared to lenalidomide–dexamethasone, showed that the median time to QoL status depreciation was longer in the investigational arm compared to the control arm [28]. In the POLLUX clinical trial, comparing Dara–Len–Dex to Len–Dex, the median time to deterioration of QoL did not differ between the two treatment arms, and the addition of daratumumab did not show any improvement in any EORTC QLQ-C30 subscales compared to Len–Dex [28,29]. The ENDEAVOR and the CASTOR clinical trials, in which the comparator arm consisted in a fixed-duration bortezomib–dexamethasone therapy, the addition of carfilzomib improved the overall QoL but did not improve the MM symptoms, while the addition of daratumumab did not add a statistically significant improvement in any of the EORTC QLQ-C30 subscales [28,30,31]. In the TOURMALINE clinical trial, the addition of ixazomib did not significantly improve the reported QoL scores compared to the control arm, with only half of the patients reporting a minimum 10-point increase in the reported scores [32].

Our study aimed to assess the perspective of Romanian MM patients on continuous therapy. This topic holds particular relevance for health systems where the majority of approved therapies are predominantly covered by the public health system, but their accessibility remains limited to major urban hematology centers. As a consequence, patients face frequent long-distance travel to receive their therapy, which disrupts their day-to-day activities.

The frequent hospital visits can negatively impact the social, professional, and financial wellbeing of the patients. More than one-third of the patients in our cohort, irrespective of their professional status, felt that continuous therapy has had a negative impact on their financial status. The Romanian healthcare system is a social system, and provides universal and complete coverage for all costs related to the care of oncology patients, including classical chemotherapy, novel therapies, supportive treatments, and management of side effects. Patients are only responsible for covering the costs of certain adjuvant medications, such as supplements or antiallergic drugs. However, the cost of transportation can be a significant burden, particularly for patients who travel across the country for ongoing treatment. Considering this, it is likely that the percentage of patients that experience financial burden in our study is mitigated by financial aid programs offered by the national MM patient association as well as by some private donations, which assist the travel costs for these patients. Furthermore, 40% of the professionally active patients reported that the therapy has had a negative impact on their professional life. This is particularly relevant because within this specific patient population, treatment-free intervals that allow patients to resume a normal lifestyle have been identified as a desirable characteristic of the therapy [27].

One-third of the patients we interviewed considered that the therapy has had negative consequences on their family life. Our study also reveals that the impact of continuous therapy extends beyond the individual, with one-third of the patients stating that their family members and their partners are also affected by the treatment schedule. Three-quarters of the interviewed patients considered that the treatment has had a negative impact on their QoL and, if given the choice, almost 60% of the patients would opt for a fixed-term therapy if provided with the same level of efficacy.

Despite these challenges, nearly all patients expressed reluctance to discontinue therapy, a sentiment possibly influenced by the fear of relapse but also by the strong patient–care provider relationships cultivated over time. Empathic care is particularly important for cancer patients who often endure substantial emotional distress. Studies have shown that higher ratings of empathy from cancer patients correlate with greater patient satisfaction and reduced emotional distress [12,13,33,34]. In our study, almost half of the patients consider that the frequent contact with their physician is psychologically comforting. This is particularly relevant for patients that have been diagnosed more than three years prior and those with lower educational status, highlighting the profound impact of long-standing, empathetic patient–physician relationships on treatment adherence and compliance. 

Despite the detrimental effects of continuous therapy on various aspects of the patients’ lives, less than one-fifth report that the frequent hospital visits are a source of anxiety. Interestingly, the lowest level of anxiety associated with the hospital visits was reported by patients diagnosed less than one year prior. This could be due to the fact that patients with more years of disease evolution are more experienced and expect potential negative news concerning their disease, while for newly diagnosed patients, the contact with the health system can be a novelty and perceived as beneficial.

Regarding therapy side effects, patients identify fatigue, bone pain, and insomnia as the most distressing. These findings are particularly significant because the severity of these symptoms depends heavily on a personal, subjective perception. Moreover, the treating physicians may not view these symptoms as critically as hematological or infectious side effects, leading to a disconnect between the patient’s and the doctor’s perspectives. As infectious complications are considered to be one of the main causes of morbidity and mortality in MM, vaccinations and prophylactic antimicrobial treatment are often associated in the therapeutic protocol [35,36], together with erythropoietin and/or granulocyte colony-stimulating factors to mitigate cytopenias. However, there is a scarcity in standardized interventions aiming to alleviate the more subjective symptoms, such as fatigue [37].

## 5. Conclusions

Continuous therapy is a relatively recent approach in MM and has been linked to improved outcomes, increased PFS, higher response rates, and more profound responses [5,21]. However, the frequent hospital visits required for this treatment can significantly disrupt patients’ daily activities. These disruptions can lead to personal, social, financial, and professional challenges, especially for patients who are still professionally active or have substantial family responsibilities. Moreover, the cumulative burden of ongoing treatment can lead to physical and psychological fatigue, making it harder for patients to adhere to the treatment over time. Accordingly, our study found that continuous therapy presents personal, social, financial, and professional challenges for a significant proportion of patients. Yet, at the same time, frequent hospital visits can provide varying degrees of psychological comfort. Regular monitoring offers a sense of security, especially when it comes to an incurable chronic disease with frequent relapses, such as MM. In our study, nearly half of the patients found frequent visits to be psychologically comforting, which contributed to a low desire to discontinue continuous therapy. Moreover, the preference for fixed-duration therapy was contingent on it offering the same level of efficacy. Despite this, nearly 40% of the patients in our cohort still favored continuous therapy even when provided with the same therapeutic efficacy. Interestingly, this preference aligns with findings from a 2023 study on chronic lymphocytic leukemia, where 40% of patients also favored continuous therapy, underscoring the importance of the perceived psychological comfort and security provided by ongoing treatment and regular monitoring [38]. These findings also highlight the importance of considering the trade-offs in relation to the duration of therapy, particularly if efficacy and safety are well established, as patients may prioritize psychological comfort and consistent monitoring over a fixed-duration approach.

Ultimately, the decision between continuous and short-term therapy requires a careful balance between achieving optimal disease control and minimizing the impact on patients’ quality of life and daily activities. A personalized approach is crucial, considering the diversity in individual values, beliefs, and disease characteristics. For example, older, retired patients or those with a more indolent disease may have different treatment preferences compared to younger, professionally active patients or those with more aggressive disease. Therefore, the choice of continuous treatment should integrate the physician’s clinical expertise with the patient’s individual preferences. Moreover, as the treatment landscape for MM continues to evolve, and more clinical experience is gained with novel drugs, there may be opportunities to adjust the dosing schedules within continuous treatment regimens. These refinements could enhance tolerance and reduce the burden of frequent hospital visits while maintaining therapeutic efficacy.

Study limitations include the single-center design, potentially affecting generalizability, although our study at Fundeni Clinical Institute involved a diverse sample across economic, social, and educational backgrounds. The questionnaire utilized in this study was translated and adapted from traditional standardized tests. It includes questions from various widely used, validated questionnaires, together with specific questions addressing our study objective. This approach not only mitigated response biases but also ensured a high completion rate of over 90% reflecting the questionnaire’s suitability for the target population, and also the external validity. More importantly, the data we gathered were not biased by the presence of a member of the medical staff during the questionnaire completion. Therefore, we believe that our study is valid and our methodology is appropriate for capturing accurate and meaningful patient-reported outcomes in this particular context.

## Figures and Tables

**Table 1:** Patient characteristics.

Sex	
Male	55.48%
Female	44.52%
Age	
<65 years	56.77%
>65 years	43.22%
Education	
Undergraduate	61.93%
Higher Education	38.06%
Occupation	
Employed	28.38%
Retired	66.45%
No occupation	5.17%
Year since diagnosis	
<1 year	18.7%
1–3 years	30.32%
>3 years	50.98%

## Data Availability

The data presented in this study are available on request from the corresponding author.
